# Musculoskeletal Diseases as the Most Prevalent Component of Multimorbidity: A Population-Based Study

**DOI:** 10.3390/jcm13113089

**Published:** 2024-05-24

**Authors:** Nina Rajovic, Slavisa Zagorac, Andja Cirkovic, Bojana Matejic, Danilo Jeremic, Radica Tasic, Jelena Cumic, Srdjan Masic, Jovana Grupkovic, Vekoslav Mitrovic, Natasa Milic, Boris Gluscevic

**Affiliations:** 1 Institute for Medical Statistics and Informatics, Faculty of Medicine, University of Belgrade, 11000 Belgrade, Serbia; nina.rajovic@med.bg.ac.rs (N.R.); andja.cirkovic@med.bg.ac.rs (A.C.); 2 Clinic for Orthopedic Surgery and Traumatology, University Clinical Center of Serbia, 11000 Belgrade, Serbia; slavisa.zagorac@gmail.com (S.Z.); grupkovicjovana@gmail.com (J.G.); 3 Faculty of Medicine, University of Belgrade, 11000 Belgrade, Serbia; danilo.jeremic@iohbb.edu.rs (D.J.); jelena.cumic@gmail.com (J.C.); glborismmm@gmail.com (B.G.); 4 Institute of Social Medicine, Faculty of Medicine, University of Belgrade, 11000 Belgrade, Serbia; bojana.matejic@med.bg.ac.rs; 5 Institute for Orthopedic Surgery “Banjica”, 11000 Belgrade, Serbia; 6 Medical School, College of Vocational Studies, 11000 Belgrade, Serbia; radica.logoped@gmail.com; 7 Department of Anesthesiology, University Clinical Center of Serbia, 11000 Belgrade, Serbia; 8 Department for Primary Health Care and Public Health, Faculty of Medicine Foca, University of East Sarajevo, 71123 East Sarajevo, Bosnia and Herzegovina; smasic@gmail.com; 9 Department for Neurology and Psychiatry, Faculty of Medicine Foca, University of East Sarajevo, 71123 East Sarajevo, Bosnia and Herzegovina; 10 Division of Nephrology and Hypertension, Mayo Clinic, Rochester, MN 55902, USA

**Keywords:** multimorbidity, musculoskeletal disorders, low back deformity, cervical deformity, osteoarthritis, hierarchical cluster analysis

## Abstract

**Background/Objectives**: Due to their high frequency, common risk factors, and similar pathogenic mechanisms, musculoskeletal disorders (MSDs) are more likely to occur with other chronic illnesses, making them a “component disorder“ of multimorbidity. Our objective was to assess the prevalence of multimorbidity and to identify the most common clusters of diagnosis within multimorbidity states, with the primary hypothesis that the most common clusters of multimorbidity are MSDs. **Methods**: The current study employed data from a population-based 2019 European Health Interview Survey (EHIS). Multimorbidity was defined as a ≥2 diagnosis from the list of 17 chronic non-communicable diseases, and to define clusters, the statistical method of hierarchical cluster analysis (HCA) was performed. **Results**: Out of 13,178 respondents, multimorbidity was present among 4398 (33.4%). The HCA method yielded six multimorbidity clusters representing the most common diagnoses. The primary multimorbidity cluster, which was prevalent among both genders, age groups, incomes per capita, and statistical regions, consisted of three diagnoses: (1) lower spine deformity or other chronic back problem (back pain), (2) cervical deformity or other chronic problem with the cervical spine, and (3) osteoarthritis. **Conclusions**: Given the influence of musculoskeletal disorders on multimorbidity, it is imperative to implement appropriate measures to assist patients in relieving the physical discomfort and pain they endure. Public health information, programs, and campaigns should be utilized to promote a healthy lifestyle. Policymakers should prioritize the prevention of MSDs by encouraging increased physical activity and a healthy diet, as well as focusing on improving functional abilities.

## 1. Introduction

Multimorbidity, which refers to the simultaneous presence of two or more chronic conditions in an individual, has gained significant attention in recent years due to its major impact on the affected individual, their family, healthcare systems, and society as a whole [1,2]. Approximately one-third of the population worldwide, including a significant fraction in low- and middle-income countries (LMICs) [3,4,5], and over half of all adults with any chronic illness have multimorbidity [1,6].

Multimorbidity can refer to many combinations of conditions, with certain disorders showing a tendency to cluster together in either remarkably similar or wildly different ways. This might be attributed to common cause elements, which may be biological, environmental, or related to pathological pathways or networks, where one condition elevates the likelihood of another [2]. Recent reviews on multimorbidity clusters have revealed significant variations among individual studies in terms of design, epidemiological and statistical methods, sampling frameworks, selection criteria, coding systems, and the identification and definition of disease cluster patterns [7,8,9]. These studies demonstrate a lack of agreement in measuring the patterns of comorbidity and multimorbidity. However, the most dominant technique used is hierarchical cluster analysis (HCA); this method is used to determine the correlation between diagnoses and is used to construct patterns of multimorbidity [7,8].

Due to their high frequency, common risk factors, and similar pathogenic mechanisms, musculoskeletal disorders (MSDs) are more likely to occur with other chronic illnesses, making them a “component disorder“ in multimorbidity. Moreover, these factors play a crucial role in the overall effects of multimorbidity, as they have been shown to diminish quality of life, raise job disability rates, and escalate treatment burden and healthcare expenses [9]. Approximately 100 million individuals in the European Union (EU) suffer from chronic musculoskeletal discomfort [10]. By 2020, there were over 500 million common cases of lower back pain globally, and this number is projected to increase to over 800 million by 2050 [11]. An estimated 8.75 million people in the UK have had treatment for osteoarthritis, which accounts for one-third of all individuals aged 45 and above [12]. Individuals with multimorbidity may have worse coping abilities and reduced health and independence due to MSDs, resulting in rapid physical and social deterioration [9]. The problem emerges when there is far less evidence of preventive measures for conditions that are constituents of prevalent clusters, such as depression or lower back pain [2]. It is, however, possible that with the prevention of depression, it may also be possible to reduce lower back pain since those with depression have a 50% higher chance of experiencing lower back pain [13]. In the case of neck pain, several studies highlighted a bidirectional relationship between primary headaches and musculoskeletal disorders. If the neck is involved in the pathogenesis, chronification process, or as a trigger for migraine episodes, it is expected that patients with migraines have a greater occurrence of musculoskeletal dysfunctions in the neck [14,15]. Nevertheless, the inquiry into the correlation and degree of interrelationships between multimorbidity and chronic back pain has received little attention.

Understanding the patterns of multimorbidity in a certain community is an essential part of creating healthcare services that are responsive to the health requirements of people with several health problems. It is crucial for to develop patient-centered approaches to prevent, diagnose, treat, and predict outcomes rather than focusing just on individual diseases. Therefore, our objective was to assess the prevalence of multimorbidity and to identify the most common clusters of diagnosis within multimorbidity states using the HCA method within the Serbian population, where our primary hypothesis was that the most common clusters of multimorbidity were MSDs.

## 2. Materials and Methods

The current study employs data from a population-based 2019 European Health Interview Survey (EHIS) [16]. This study was conducted over a three-month period (October–December) in 2019, following the guidelines of the European Health Survey—the third wave. These guidelines specify that data collection in the field should last for a minimum of three months, with at least one month falling within the autumn season (September–December). In order to obtain a nationally representative survey, the Institute of Public Health of Serbia “Dr Milan Jovanovic Batut” collaborated with the Statistical Office of the Republic of Serbia and the Ministry of Health of the Republic of Serbia. This study was conducted in accordance with the Declaration of Helsinki and approved by the Institutional Review Board of the Institute of Public Health of Serbia “Dr Milan Jovanovic Batut” (protocol code 1982/1, 14 April 2022). In order to obtain a nationally representative sample, a stratified two-stage cluster sampling method was used. The process of stratification was carried out based on the following four areas in Serbia: Vojvodina, Šumadija and Western Serbia, Southern and Eastern Serbia, and the Belgrade region, as well as the kind of habitation (urban or other). The first phase included the use of cluster sampling methods to randomly choose census districts with a probability proportionate to their respective sizes. In the second phase, a random sample of households was selected with an equal probability. A total of 5114 homes, representing a response rate of 85.23%, were included in the sample out of the 6000 households that were invited. The inclusion criteria were individuals aged 15 years and above, residing in non-institutional (private) homes within the area of the Republic of Serbia, and representing the typical population. Individuals residing in communal residences were excluded from the study. Further details of the Serbian EHIS 2019 sampling processes, sample size calculation, and criteria for including and excluding the population may be found elsewhere [17].

According to the available variables from the survey, multimorbidity was defined as ≥2 diagnoses from the list of 17 chronic non-communicable diseases: (1) hypertension; (2) lower spine deformity or other chronic back problem (back pain); (3) cervical deformity or other chronic problem with the cervical spine; (4) high blood fat (cholesterol); (5) osteoarthritis; (6) coronary artery disease or angina pectoris; (7) an allergy (excluding allergic asthma); (8) diabetes mellitus; (9) depression (or chronic anxiety); (10) renal disorders; (11) chronic bronchitis, COPD (chronic obstructive pulmonary disease), emphysema; (12) urinary incontinence; (13) asthma (including allergic asthma); (14) stroke (cerebral bleeding or thrombosis) or chronic consequences of a stroke; (15) myocardial infarction or chronic consequences of the myocardial infarction; (16) malignancies; and (17) liver cirrhosis.

In order to define clusters, HCA was performed. HCA was performed by variables using Ward’s standard method (Euclidean distance) and variable standardization via the z score. The distribution of multimorbidity, as well as clustering, was evaluated according to gender (male, female), age groups (≤65 years, 65+ years), and household monthly income per person divided into quintiles, with the 1st quintile being the lowest income and the 5th quintile representing the highest income level, and four statistical regions in Serbia (Vojvodina, Belgrade, Šumadija and Western Serbia, South and Eastern Serbia).

The numerical variable (age) was presented with the arithmetic mean and standard deviation. Categorical variables were described with absolute and relative numbers in percentages. Student’s *t*-test was performed to test the differences between the two groups according to the numerical variable with normal distribution, while the Chi-square test was used to compare two independent groups according to categorical variables. All statistical methods were considered significant if *p* ≤ 0.05. Analysis was performed using the IBM SPSS software (IBM SPSS Statistics for Windows, Version 21.0, IBM Corp, Armonk, NY, USA).

## 3. Results

The study comprised a total of 13,178 participants, with a mean age of 50.70 ± 19.06 years. Multimorbidity was present among 4398 (33.4%) of all respondents, 1852 (28.8%) among men and 2546 (37.8%) among women. The occurrence of multimorbidity was much higher in females (*p* < 0.001).

**Figure 1:**
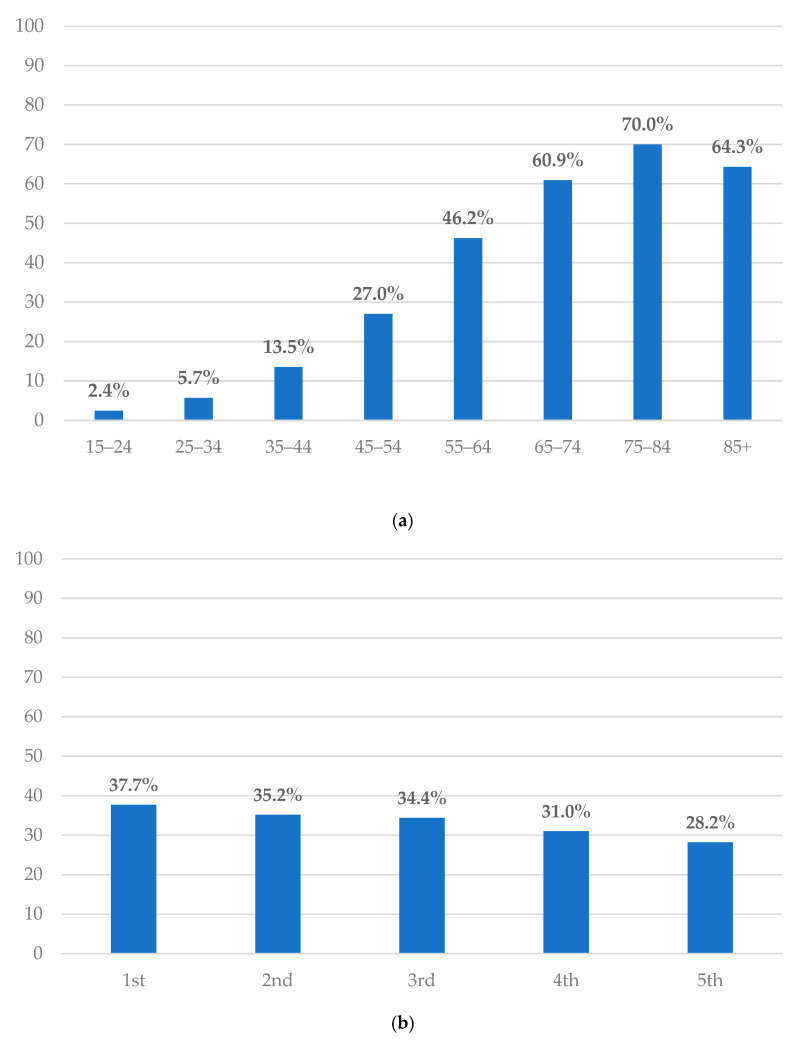

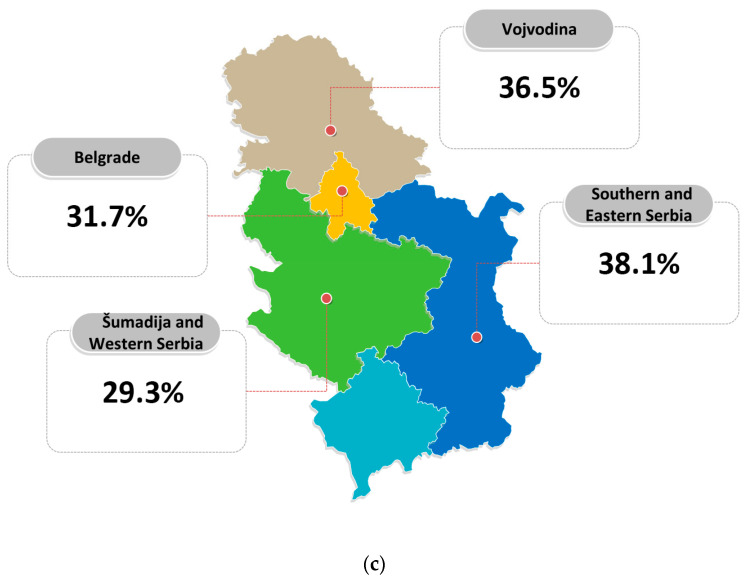
(**a**) Multimorbidity distribution categorized by age groups; (**b**) multimorbidity distribution based on monthly income per capita; (**c**) multimorbidity distribution in four statistical regions of Serbia.

Figure 1a illustrates the distribution of multimorbidity categorized by age group. The prevalence of multimorbidity was highest among those aged 75–84 (70.0%), followed by those aged 85 years and beyond (64.3%), and individuals aged 65 to 74 years (60.9%). Residents older than 65 years had a considerably higher prevalence of multimorbidity compared to those aged 65 years or younger (21.5% vs. 63.9%, *p* < 0.001) (Table 1).

The prevalence of multimorbidity varied significantly based on education level, with the greatest frequency seen among those with a primary educational level (*p* < 0.001) (Table 1).

Figure 1b displays the distribution of multimorbidity based on the monthly income per capita, expressed as quintiles. The prevalence of multimorbidity was the highest among those in the first income quintile group and lowest among those in the fifth quintile group. The prevalence of multimorbidity experienced a significant decline from the lowest socioeconomic status group (first quintile) to the highest socioeconomic status group (fifth quintile) (*p* < 0.001) (Table 1).

Figure 1c displays the distribution of multimorbidity in four statistical regions of Serbia. There was a significant difference in the occurrence of multimorbidity across different areas. The southern and eastern regions of Serbia had the greatest incidence of multimorbidity, followed by the Vojvodina region (*p* < 0.001).

### Multimorbidity Clustering

**Figure 2:**
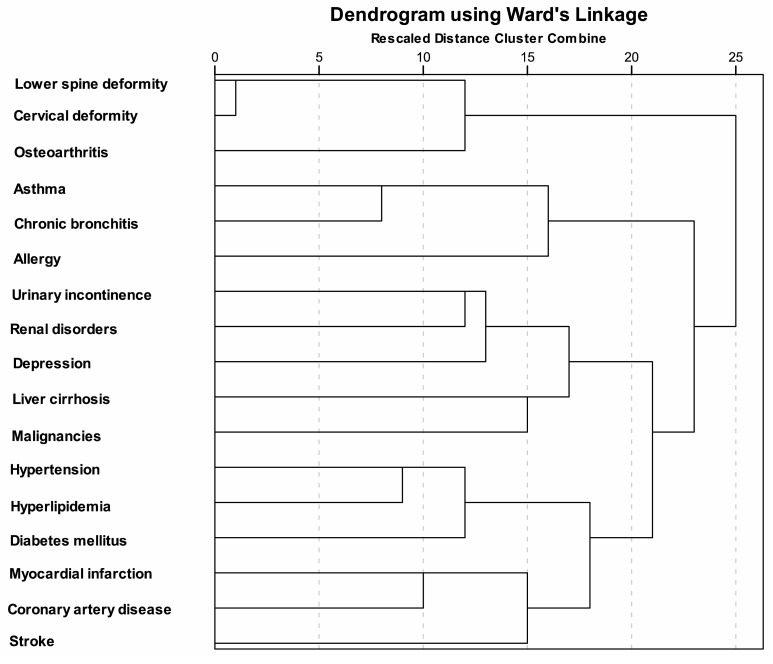
Dendrogram of most common clusters of multimorbidity obtained by HCA method.

The HCA method yielded six clusters representing the most common diagnoses. The diagnosis for each cluster can be found in Table 2. The most prevalent cluster included the following diagnoses: (1) lower spine deformity or other chronic back problems (back pain), (2) cervical deformity or other chronic problems with the cervical spine, and (3) osteoarthritis. The following cluster included (1) asthma (including allergic asthma), (2) chronic bronchitis, COPD, emphysema, and (3) allergies (excluding allergic asthma). The third cluster covered (1) hypertension, (2) diabetes mellitus, and (3) high blood fat (cholesterol), while the last cluster comprised (1) myocardial infarction or chronic consequences of the myocardial infarction, (2) coronary artery disease or angina pectoris, and (3) stroke (cerebral bleeding or thrombosis) or chronic consequences of a stroke. In Figure 2. a dendrogram of the most common clusters of multimorbidity obtained by the HCA method is shown.

The clusters of diagnosis according to gender, age, income, and statistical regions in Serbia produced comparable outcomes. The primary multimorbidity cluster, which was prevalent among both genders, age groups, incomes per capita, and statistical regions, consisted of the following three diagnoses: (**1**) **lower spine deformity or other chronic back problem** (**back pain**)**,** (**2**) **cervical deformity or other chronic problem with the cervical spine, and** (**3**) **osteoarthritis.** Clusters of common diagnoses according to the sociodemographic characteristics of the study population obtained by the HCA method are presented in Supplementary Materials Table S1.

## 4. Discussion

The current research findings indicated that when using the HCA clustering methodology, low and cervical back problems and osteoarthritis emerged as the most prominent multimorbidity cluster of disorders. We were able to identify six clinically valid clusters among the seventeen most frequent chronic conditions in the population of Serbia.

The results of the study conducted by Schäfer et al. [18] show that the chronic disease that seemed to be the most significant mediator of linkages between other chronic disorders was chronic lower back pain, which also happened to be the condition that had the greatest overall number of associations. In their prior study, Schäfer et al. [19] revealed that the incidence of chronic lower back pain increased beyond what would be expected in proportion to the number of comorbidities an individual had. Physicians frequently encounter chronic low back pain as a secondary diagnosis when multimorbid patients seek medical attention for unrelated concerns. For instance, 71% of patients with back pain did not visit their physician for this reason, according to Waxman et al. [20]. Additionally, Scherer et al. [21] indicated that patients with multimorbidity are significantly impacted by lower back issues. Their study results showed that 79% of females and 83% of males included were diagnosed with spondylosis, intervertebral disc disorders, spinal osteochondrosis, or other degenerative back problems. The high prevalence of MSDs in relation to other chronic conditions as part of multimorbidity is influenced by a multitude of factors. For instance, research has demonstrated that nearly one-third of English primary care patients aged 45 and above who report having a major long-term condition also have a musculoskeletal condition. Furthermore, nearly 50% of individuals aged over 65 with a heart, lung, or mental health condition also experience an MSD [22]. Among individuals already afflicted with heart disease, diabetes, COPD, or cancer, osteoarthritis, and back pain are the prevailing comorbidities in the most deprived populations [1]. The results of our study show a similarity; in both males and females, both age groups, all income quantiles, and statistical regions in Serbia, the most common multimorbidity cluster consisted of three diagnoses: lower spine deformity or other chronic back problem (back pain), cervical deformity or other chronic problem with the cervical spine, and osteoarthritis.

It has been reported that adults with multiple chronic conditions and concurrent musculoskeletal pain tend to report increased levels of disability, mental health issues, reduced work capacity, physical inactivity, and obesity [23,24,25,26,27,28,29,30,31,32]. Given the intricate clinical presentation and the combined impact of all these conditions, it is unsurprising that individuals with multimorbidity also experience a heightened perception of illness, diminished pain self-efficacy, and reduced overall health [30]. Individuals with lower socioeconomic status are more likely to experience negative health outcomes, while individuals with a lower level of education are more likely to have multiple chronic health conditions [33,34]. The findings of our study provide evidence for this claim, as the occurrence of multimorbidity differs significantly depending on the level of education and economic status. The highest frequency was observed among individuals with a primary level of education and those who were most economically deprived. Furthermore, our study findings indicate that HCA and EFA methodologies reveal a prominent cluster of diseases, namely low spine and cervical deformity and osteoarthritis, among individuals belonging to the lowest socioeconomic class. A study conducted by Mujica-Mota et al. [35] revealed that osteoarthritis and back pain are two of the most prevalent conditions that significantly decrease an individual’s health-related quality of life. These conditions also have a widespread impact on the overall population due to their high occurrence [35]. Furthermore, there is a substantial influence on financial well-being. Due to work impairment and higher personal expenses, 73% of individuals with severe arthritis face challenges in maintaining their financial stability in relation to their income. However, only 6% of individuals without functional limitations experience similar difficulties [36].

In contrast to the extensive number of trials evaluating treatments for back pain and osteoarthritis, there is insufficient evidence regarding prevention, especially primary prevention. Additionally, there are worldwide disparities between the available evidence and the actual implementation of medical practices. This becomes evident in the inadequate use of recommended initial treatments and the excessive reliance on imaging, rest, opioids, spinal injections, and surgery [37]. Individuals suffering from osteoarthritis have also highlighted an insufficient acknowledgment of their conditions within healthcare services and society in general. This lack of recognition may stem from a prioritization of conditions with higher mortality rates rather than those that primarily cause morbidity and decrease quality of life. This may be due to a nihilistic belief that nothing can be done for individuals with MSDs and that arthritis is an unavoidable consequence of aging. Additionally, challenges in measuring musculoskeletal health outcomes arise from the absence of biomarkers or simple tests used to monitor musculoskeletal health [9]. Over the past few years, significant suggestions in national clinical practice guidelines have been recommended for the treatment of lower back pain. There is now a stronger focus on self-management, physical and psychological therapies, and certain types of complementary medicine, while less importance is given to pharmacological and surgical treatments. Guidelines promote the use of active treatments that target psychosocial factors and prioritize enhancing functional improvement [37]. According to the World Health Organization (WHO), preventative measures that should be considered include physical therapies aimed at enhancing muscular strength and mobility, psychological and social assistance provided to aid individuals in pain management and the restoration of their engagement in enjoyable activities, the implementation of strategies to minimize physical strain during work-related activities, and the adoption of lifestyle modifications encompassing increased physical activity, a nutritious diet, and improved sleep patterns [38].

Having good musculoskeletal health is essential for maintaining independence when dealing with multiple long-term conditions. It is imperative to acknowledge and tackle it as an integral component of multimorbidity. The metrics and tools designed for multimorbidity management programs should track outcomes that are related to musculoskeletal health, such as pain and its consequences, as well as functional capacity. The regular collection, publication, and utilization of reliable data on MSDs and multimorbidity in various sectors, such as public health, healthcare, and related systems, should be used to determine the extent and requirements of individuals with multimorbidity and to enhance the delivery of services and activities aimed at improving their quality. Presently, regularly gathered data occasionally fail to acknowledge the simultaneous presence of MSDs, resulting in significant underestimations of the prevalence of multimorbidity, not to mention their influence. Local government and public health teams, as well as healthcare commissioners, payers, and providers, should analyze, categorize, and comprehend the needs and demands of individuals residing with MSDs and multimorbidity within their population.

### Strengths and Limitations

This study’s main strength is the use of a representative sample of the Serbian population. Furthermore, one of the main limitations of the methodology used in this study could be seen as a strength. Namely, according to the EHIS methodology, we used 17 chronic conditions for the analysis of multimorbidity. Although not exhaustive, this list was uniformly used in all counties performing EHIS; therefore, our results could be comparable to those of other countries. However, the presence of chronic illnesses was based on self-reported data, which potentially introduces the recall bias. This bias resulting from variations in detection, diagnosis, and memory is likely more pronounced in diseases with milder symptoms, such as hypertension or diabetes, compared to diseases with more severe symptoms, such as myocardial infarction, stroke, urine incontinence, or COPD. Another limitation of this study is the use of the HCA method, which assigns each diagnosis to only a single cluster. This limitation suggests the need for additional research using alternative clustering methods to explore potential patterns of multimorbidity.

## 5. Conclusions

Given the influence of musculoskeletal disorders on multimorbidity, it is imperative to implement appropriate measures to assist patients in relieving the physical discomfort and pain they endure. Public health information, programs, and campaigns should be utilized to promote a healthy lifestyle. Policymakers should prioritize the prevention of MSDs by encouraging increased physical activity and a healthy diet, as well as focusing on improving functional abilities.

## Figures and Tables

**Table 1:** Distribution of multimorbidity according to sociodemographic characteristics.

Variable	Multimorbidity		*p*
	No (*n* = 8778)	Yes (*n* = 4400)	
**Gender, *n* (%)**			
Male	4571 (71.2)	1852 (28.8)	**<0.001**
Female	4197 (62.2)	2546 (37.8)	
**Age, *n* (%)**			
<65	7433 (78.5)	2031 (21.5)	**<0.001**
65+	1335 (36.1)	2367 (63.9)	
**Educational level, *n* (%)**			
Primary	1826 (51.8)	1699 (48.2)	**<0.001**
Secondary	5198 (71.3)	2089 (28.7)	
Tertiary	1744 (74.1)	610 (25.9)	
**Income quintiles, *n* (%)**			
1st	1691 (62.3)	1024 (37.7)	**<0.001**
2nd	1747 (64.8)	947 (35.2)	
3rd	1746 (65.6)	915 (34.4)	
4th	1819 (69.0)	818 (31.0)	
5th	1765 (71.8)	694 (28.2)	
**Statistical regions, *n* (%)**			
Belgrade region	2087 (68.3)	968 (31.7)	**<0.001**
Vojvodina region	1881 (63.5)	1079 (36.5)	
Šumadija and Western Serbia	2993 (70.7)	1238 (29.3)	
Southern and Eastern Serbia	1807 (61.9)	1113 (38.1)	

**Table 2:** Clusters of multimorbidity according to HCA method.

Cluster No.	HCA
**1st**	(1) Lower spine deformity or other chronic back problem (back pain); (2) Cervical deformity or other chronic problem with the cervical spine; (3) Osteoarthritis.
**2nd**	(1) Asthma (including allergic asthma); (2) Chronic bronchitis, COPD, Emphysema; (3) Allergy (excluding allergic asthma).
**3rd**	(1) Urinary incontinence; (2) Renal disorders; (3) Depression.
**4th**	(1) Liver cirrhosis; (2) Malignancies.
**5th**	(1) Hypertension; (2) Hyperlipidemia; (3) Diabetes mellitus.
**6th**	(1) Myocardial infarction or chronic consequences of the myocardial infarction; (2) Coronary artery disease or angina pectoris; (3) Stroke (cerebral bleeding or thrombosis) or chronic consequences of a stroke.

## Data Availability

The data that support the findings of this study are available on request from the corresponding author.

## Supplementary Materials

The following supporting information can be downloaded at: https://www.mdpi.com/article/10.3390/jcm13113089/s1, Table S1: Clusters with lists of diagnosis according to HCA method by gender; Table S2: Clusters with lists of diagnosis according to HCA method by age groups; Table S3: Clusters with lists of diagnosis according to HCA method by income; Table S4: Clusters with lists of diagnosis according to HCA method by statistical regions in Serbia.
