# Supplementary material for: Musculoskeletal Diseases as the Most Prevalent Component of Multimorbidity: A Population-Based Study
Source: J Clin Med. 2024 May 24;13(11):3089. doi: 10.3390/jcm13113089 (PMC11172850; doi:10.3390/jcm13113089)
Supplement: Supplementary file 1 [file jcm-13-03089-s001.zip › jcm-2992080-supplementary.pdf]

Table S1. Clusters with lists of diagnosis according to HCA method by gender

Cluster No	Gender	
	Men	Women
1st	1) Lower spine deformity or other chronic back problem (back pain)<br>2) Cervical deformity or other chronic problem with the cervical spine<br>3) Osteoarthritis	1) Lower spine deformity or other chronic back problem (back pain)<br>2) Cervical deformity or other chronic problem with the cervical spine<br>3) Osteoarthritis
2nd	1) Asthma (including allergic asthma)<br>2) Chronic bronchitis, COPD, Emphysema	1) Hypertension<br>2) High blood fat (cholesterol)<br>3) Diabetes mellitus
3rd	1) Hypertension<br>2) High blood fat (cholesterol)<br>3) Diabetes mellitus	1) Myocardial infarction or chronic consequences of the myocardial infarction<br>2) Coronary artery disease or angina pectoris<br>3) Stroke (cerebral bleeding or thrombosis) or chronic consequences of the stroke
4th	1) Myocardial infarction or chronic consequences of the myocardial infarction<br>2) Coronary artery disease or angina pectoris<br>3) Stroke (cerebral bleeding or thrombosis) or chronic consequences of the stroke	1) Asthma (including allergic asthma)<br>2) Chronic bronchitis, COPD, Emphysema<br>3) Allergy (excluding allergic asthma)
5th	1) Urinary incontinence<br>2) Renal disorders<br>3) Depression	1) Urinary incontinence<br>2) Renal disorders<br>3) Depression
6th	1) Allergy<br>2) Liver cirrhosis<br>3) Malignancies	1) Liver cirrhosis<br>2) Malignancies

Table S2. Clusters with lists of diagnosis according to HCA method by age groups

Cluster No	Age group	
	**≤65 years**	**65+ years**
1st	1) Lower spine deformity or other chronic back problem (back pain)<br>2) Cervical deformity or other chronic problem with the cervical spine<br>3) Osteoarthritis	1) Lower spine deformity or other chronic back problem (back pain)<br>2) Cervical deformity or other chronic problem with the cervical spine<br>3) Osteoarthritis
2nd	1) Asthma (including allergic asthma)<br>2) Chronic bronchitis, COPD, Emphysema<br>3) Allergy	1) Asthma (including allergic asthma)<br>2) Chronic bronchitis, COPD, Emphysema
3rd	1) Urinary incontinence<br>2) Renal disorders<br>3) Depression<br>4) Liver cirrhosis<br>5) Malignancies	1)Urinary incontinence<br>2)Renal disorders<br>3)Depression
4th	1) Hypertension<br>2) High blood fat (cholesterol)<br>3) Diabetes mellitus	1) Allergy (excluding allergic asthma)<br>2) Liver cirrhosis<br>3) Malignancies
5th	1) Myocardial infarction or chronic consequences of the myocardial infarction<br>2) Coronary artery disease or angina pectoris<br>3) Stroke (cerebral bleeding or thrombosis) or chronic consequences of the stroke	1) Hypertension<br>2) High blood fat (cholesterol)<br>3) Diabetes mellitus
6th		1) Myocardial infarction or chronic consequences of the myocardial infarction<br>2) Coronary artery disease or angina pectoris<br>3) Stroke (cerebral bleeding or thrombosis) or chronic consequences of the stroke

Table S3. Clusters with lists of diagnosis according to HCA method by income

Cluster No	1st quintile
1st	1) Lower spine deformity or other chronic back problem (back pain)<br>2) Cervical deformity or other chronic problem with the cervical spine<br>3) Osteoarthritis
2nd	1) Asthma (including allergic asthma)<br>2) Chronic bronchitis, COPD, Emphysema<br>3) Allergy<br>4) Cirrhosis
3rd	1) Urinary incontinence<br>2) Renal disorders<br>3) Depression<br>4) Stroke (cerebral bleeding or thrombosis) or chronic consequences of the stroke<br>5) Malignancies
4th	1) Myocardial infarction or chronic consequences of the myocardial infarction<br>2) Coronary artery disease or angina pectoris
5th	1) Hypertension<br>2) High blood fat (cholesterol)<br>3) Diabetes mellitus
**Cluster No**	**2nd quintile**
1st	1) Lower spine deformity or other chronic back problem (back pain)<br>2) Cervical deformity or other chronic problem with the cervical spine<br>3) Osteoarthritis
2nd	1) Asthma (including allergic asthma)<br>2) Chronic bronchitis, COPD, Emphysema
3rd	1) Liver cirrhosis<br>2) Malignancies
4th	1) Urinary incontinence<br>2) Renal disorders<br>3) Depression<br>4) Allergy
5th	1) Myocardial infarction or chronic consequences of the myocardial infarction<br>2) Stroke (cerebral bleeding or thrombosis) or chronic consequences of the stroke
6th	1) Coronary artery disease or angina pectoris<br>2) Hypertension<br>3) High blood fat (cholesterol)<br>4) Diabetes mellitus
**Cluster No**	**3rd quintile**
1st	1) Lower spine deformity or other chronic back problem (back pain)<br>2) Cervical deformity or other chronic problem with the cervical spine<br>3) Osteoarthritis
2nd	1) Asthma (including allergic asthma)<br>2) Chronic bronchitis, COPD, Emphysema<br>3) Allergy
3rd	1) Myocardial infarction or chronic consequences of the myocardial infarction<br>2) Coronary artery disease or angina pectoris
4th	1) Hypertension<br>2) High blood fat (cholesterol)<br>3) Diabetes mellitus
5th	1) Stroke (cerebral bleeding or thrombosis) or chronic consequences of the stroke<br>2) Liver cirrhosis
6th	1) Urinary incontinence<br>2) Renal disorders<br>3) Depression<br>4) Malignancies
**Cluster No**	**4th quintile**
1st	1) Lower spine deformity or other chronic back problem (back pain)<br>2) Cervical deformity or other chronic problem with the cervical spine<br>3) Osteoarthritis
2nd	1) Hypertension<br>2) High blood fat (cholesterol)<br>3) Diabetes mellitus
3rd	1) Myocardial infarction or chronic consequences of the myocardial infarction<br>2) Coronary artery disease or angina pectoris

	3) Stroke (cerebral bleeding or thrombosis) or chronic consequences of the stroke
4th	1) Asthma (including allergic asthma)<br>2) Chronic bronchitis, COPD, Emphysema<br>3) Allergy
5th	1) Liver cirrhosis<br>2) Malignancies
6th	1) Urinary incontinence<br>2) Renal disorders<br>3) Depression
**Cluster No**	**5th quintile**
1st	1) Lower spine deformity or other chronic back problem (back pain)<br>2) Cervical deformity or other chronic problem with the cervical spine
2nd	1) Urinary incontinence<br>2) Renal disorders<br>3) Osteoarthritis<br>4) Depression
3rd	1) Liver cirrhosis<br>2) Malignancies
4th	1) Hypertension<br>2) High blood fat (cholesterol)<br>3) Diabetes mellitus
5th	1) Myocardial infarction or chronic consequences of the myocardial infarction<br>2) Coronary artery disease or angina pectoris<br>3) Stroke (cerebral bleeding or thrombosis) or chronic consequences of the stroke
6th	1) Asthma (including allergic asthma)<br>2) Chronic bronchitis, COPD, Emphysema<br>3) Allergy

Table S4. Clusters with lists of diagnosis according to HCA method by statistical regions in Serbia

Cluster No	Belgrade
1st	1) Lower spine deformity or other chronic back problem (back pain)<br>2) Cervical deformity or other chronic problem with the cervical spine<br>3) Osteoarthritis
2nd	1) Myocardial infarction or chronic consequences of the myocardial infarction<br>2) Coronary artery disease or angina pectoris
3rd	1) Hypertension<br>2) High blood fat (cholesterol)<br>3) Diabetes mellitus
4th	1) Asthma (including allergic asthma)<br>2) Chronic bronchitis, COPD, Emphysema<br>3) Allergy
5th	1) Liver cirrhosis<br>2) Malignancies
6th	1) Urinary incontinence<br>2) Renal disorders<br>3) Stroke (cerebral bleeding or thrombosis) or chronic consequences of the stroke<br>4) Depression
**Cluster No**	**Vojvodina**
1st	1) Lower spine deformity or other chronic back problem (back pain)<br>2) Cervical deformity or other chronic problem with the cervical spine<br>3) Osteoarthritis
2nd	1) Asthma (including allergic asthma)<br>2) Chronic bronchitis, COPD, Emphysema<br>3) Allergy
3rd	1) Urinary incontinence<br>2) Depression<br>3) Renal disorders<br>4) Liver cirrhosis<br>5) Malignancies
4th	1) Hypertension<br>2) High blood fat (cholesterol)<br>3) Diabetes mellitus
5th	1) Myocardial infarction or chronic consequences of the myocardial infarction<br>2) Coronary artery disease or angina pectoris<br>3) Stroke (cerebral bleeding or thrombosis) or chronic consequences of the stroke
**Cluster No**	**Šumadija and Western Serbia**
1st	1) Lower spine deformity or other chronic back problem (back pain)<br>2) Cervical deformity or other chronic problem with the cervical spine<br>3) Osteoarthritis
2nd	1) Coronary artery disease or angina pectoris<br>2) Hypertension<br>3) High blood fat (cholesterol)<br>4) Diabetes mellitus
3rd	1) Stroke (cerebral bleeding or thrombosis) or chronic consequences of the stroke<br>2) Depression<br>3 Myocardial infarction or chronic consequences of the myocardial infarction
4th	1) Asthma (including allergic asthma)<br>2) Chronic bronchitis, COPD, Emphysema
5th	1) Urinary incontinence<br>2) Renal disorders<br>3) Allergy<br>4) Liver cirrhosis<br>5) Malignancies
**Cluster No**	**Eastern and Southern Serbia**
1st	1) Lower spine deformity or other chronic back problem (back pain)<br>2) Cervical deformity or other chronic problem with the cervical spine<br>3) Osteoarthritis
2nd	1) Asthma (including allergic asthma)<br>2) Chronic bronchitis, COPD, Emphysema
3rd	1) Urinary incontinence

	2) Renal disorders<br>3) Depression
4th	1) Allergy<br>2) Liver cirrhosis<br>3) Malignancies
5th	1) Hypertension<br>2) High blood fat (cholesterol)<br>3) Coronary artery disease or angina pectoris<br>4) Diabetes mellitus
6th	1) Myocardial infarction or chronic consequences of the myocardial infarction<br>2) Stroke (cerebral bleeding or thrombosis) or chronic consequences of the stroke
